# *Phlebotomus perniciosus* response to volatile organic compounds of dogs and humans

**DOI:** 10.1371/journal.pntd.0012787

**Published:** 2024-12-30

**Authors:** Marcos Antonio Bezerra-Santos, Valeria Zeni, Onofrio Marco Pistillo, Stefano Bedini, Ilaria D’Isita, Giovanni Benelli, Giacinto Salvatore Germinara, Petr Volf, Domenico Otranto

**Affiliations:** 1 Department of Veterinary Medicine, University of Bari, Bari, Italy; 2 Department of Agriculture, Food and Environment, University of Pisa, Pisa, Italy; 3 Department of Agricultural Sciences, Food, Natural Resources and Engineering, University of Foggia, Foggia, Italy; 4 Department of Parasitology, Faculty of Science, Charles University, Staré Město, Czech Republic; 5 Department of Veterinary Clinical Sciences, City University of Hong Kong, Hong Kong, China; Instituto Oswaldo Cruz, BRAZIL

## Abstract

The olfactory response of insect vectors such as phlebotomine sand flies is a key facet for investigating their interactions with vertebrate hosts and associated vector-borne pathogens. Such studies are mainly performed by assessing the electrophysiological response and the olfactory behaviour of these arthropods towards volatile organic compounds (VOCs) produced by hosts. Nonetheless, few studies are available for species of the subgenera *Lutzomyia* and *Nyssomyia* in South America, leaving a void for Old World sand fly species of the genus *Phlebotomus*. In this study, we evaluated the olfactory responses of *Phlebotomus perniciosus*, one of the most important vectors of *Leishmania infantum* in the Old World. To test the *P*. *perniciosus* behavioural response to VOCs, 28 compounds isolated from humans and dogs were assessed using electrophysiological (i.e., electroantennogram, EAG) and behavioural assays (i.e., Y-tube olfactometer). In the EAG trials, 14 compounds (i.e., acetic acid, nonanoic acid, 2-propanol, 2-butanol, pentanal, hexanal, nonanal, *trans*-2-nonenal, decanal, myrcene, *p*-cymene, verbenone, 2-ethyl-1-hexanol, and acetonitrile) elicited high antennal responses (i.e., ≥ 0.30 mV) in female sand flies, being those VOCs selected for the behavioural assays. From the 14 compounds tested in the Y-tube olfactometer, nonanal was significantly attractive for *P*. *perniciosus* females, whereas myrcene and *p*-cymene were significantly repellents (*p* < 0.05). The attraction indexes varied from 0.53 for nonanal (i.e., most attractive) to -0.47 to *p*-cymene (i.e., most repellent). Overall, our results shed light on the role of olfactory cues routing host seeking behaviour in *P*. *perniciosus*, with implications to develop sustainable sand fly monitoring as well as control in leishmaniasis endemic areas.

## Author summary

The way insects respond to host odours is a key aspect for understanding how they interact with their hosts and associated pathogens. Such studies are mainly performed by assessing the attraction or repellence of these arthropods towards host emitted volatile organic compounds (VOCs). In this study, we investigated how *Phlebotomus perniciosus* reacts to host odours. For this, 28 compounds isolated from humans and dogs were assessed by antennal stimulus and odour choice by sand flies. Fourteen compounds elicited high antennal responses in female sand flies, being those VOCs selected for the behavioural assays. From the 14 compounds tested in the Y-tube olfactometer, nonanal was significantly attractive for *P*. *perniciosus* females, whereas myrcene and *p*-cymene acted as repellents (*p* < 0.05). Data herein obtained highlight the role of olfactory cues routing host seeking behaviour in *P*. *perniciosus*, with implications to develop sustainable sand fly monitoring and control, in leishmaniasis endemic areas.

## Introduction

Phlebotomine sand flies (Diptera: Psychodidae) are hematophagous insects that act as vectors of pathogens of significant human and veterinary relevance. Among the infectious agents transmitted by these arthropods, *Leishmania* spp. are parasitic protozoa of major concern due to their ability to cause disease in many animal species, including humans. These parasites have a complex life cycle involving both domestic and wild animals worldwide. The perpetuation of the transmission of *Leishmania* spp. protozoa is guaranteed by their vectors, which are *Phlebotomus* spp. in the Old World, and mainly species of the genus *Lutzomyia* in the Americas [1,2]. *Phlebotomus perniciosus* Newsted, 1911 is the main vector of *Leishmania infantum* Nicolle, 1908, the causative agent of visceral leishmaniasis in the Mediterranean basin where domestic dogs act as reservoirs [3,4].

The interplay amongst sand fly vectors, susceptible hosts, and the environment is pivotal for understanding the ecology of the pathogens they may transmit. In this context, vectorial capacity and host seeking preference of sand flies play an essential role. Indeed, studies investigating the interaction among sand fly species and their hosts have been performed worldwide, using methods such as detection of the host blood meal [5–8], and host-choice assessments in laboratory conditions [9,10]. In addition, the role of olfactory cues, such as volatile organic compounds (VOCs) emitted by hosts, has gained attention in studies of insect vector behaviour, particularly in mosquitoes (Diptera: Culicidae) [reviewed in 11] and triatomine bugs (Hemiptera: Reduviidae) [12,13]. Nevertheless, for sand flies, only few studies were focused on species within the subgenera *Lutzomyia* and *Nyssomyia* in South America, by testing VOCs from humans, dogs and foxes [14–21]. To date, the effects of VOCs have not been rigorously assessed for *Phlebotomu*s species, as studies on the attractiveness of *P*. *perniciosus* and *P*. *perfiliewi* Parrot, 1930 did not analytically test these compounds [22].

Insects perceive host odours by olfactory receptors located mainly in antennal sensilla. Behaviourally active compounds can be identified using the electroantennographic technique (EAG), which measures the electrical signals associated with olfaction. The EAG response which represents the antennal olfactory sensitivity to a tested compound, often has ecological significance [23,24]. Therefore, this technique is useful to screen and select suitable VOCs, which can be further tested in behavioural olfactometric experiments (e.g., flight tunnel and field/semi-field tests) to assess the insect responses [25]. The evaluation of insects´ capability to perceive VOCs emitted by hosts and understanding behavioural effects are crucial steps for identification of attractive or repellent molecules that can be used to develop suitable monitoring and eco-friendly control tools.

In this study, we hypothesize that the VOCs associated with human and canine hosts may play a significant role in the host-seeking behaviour of *P*. *perniciosus*. We examined the olfactory responses of *P*. *perniciosus* females to selected human and dog VOCs, exploring their perception and the behavioural responses they elicit. We also discuss the potential application of these VOCs for sand fly monitoring and control.

## Methods

### Insects

*Phlebotomus perniciosus* sand flies were reared at the Department of Veterinary Medicine of the University of Bari according to previously described procedures [26]. Newly emerged males and females were transferred to fine tulle cages (30 x 30 x 30 cm) and provided with a 50% sucrose solution as food. Females, 6–12 days old, were used for the electrophysiological and behavioural tests described below.

### Odour stimuli

The tested compounds were selected from a bouquet of VOCs (S1 Table) previously identified from humans and dogs [18,27–29] representing different chemical classes (i.e., aliphatic alcohols, aldehydes, acids, hydrocarbons, terpenoids, and other compounds) (Table 1). The compounds were selected based on their abundance on the hosts, considering data observed in previous studies [18,27–29]. To prevent rapid evaporation of these compounds, they were dissolved in mineral light oil (Sigma-Aldrich, Milan, Italy). For each compound, a 100 μg/μL solution was prepared. To obtain EAG dose-response curves, mineral oil solutions of hexanal (0.01, 0.1, 1, 10, 100, and 200 μg/μL), were used as a reference stimulus since this compound has been proven to trigger high antennal response in insects [30,31]. Solutions were stored at -20°C until needed.

**Table 1:** Mean (±SE) EAG responses (mV) of female *P*. *perniciosus* to 28 volatile organic compounds (VOCs) identified from head-space samples of human and dog hairs.

Class compounds	Absolute EAG response in mV (mean ±SE)
*Aliphatic acids*	
Acetic acid	0.47±0.02
Heptanoic acid	0.24±0.01
Nonanoic acid	0.35±0.01
*Aliphatic alcohols*	
2-Propanol	0.32±0.01
2-Butanol	0.30±0.02
1-Octanol	0.22±0.02
3-Octanol	0.17±0.02
*Aliphatic aldehydes*	
Pentanal	0.81±0.06
Hexanal	0.96±0.07
Nonanal	0.39±0.07
*trans*-2-Nonenal	0.72±0.04
Decanal	0.33±0.02
*Aliphatic hydrocarbons*	
Heptane	0.27±0.01
Decane	0.27±0.01
Undecane	0.23±0.01
Pentadecane	0.26±0.01
Hexadecane	0.29±0.01
Heptadecane	0.22±0.01
Octadecane	0.23±0.01
Nonadecane	0.28±0.01
*Terpenes*	
α-Pinene	0.23±0.01
Myrcene	0.34±0.01
*p*-Cymene	0.30±0.01
Verbenone	0.39±0.01
*Others*	
2-Ethyl-1-hexanol	0.37±0.02
Acetonitrile	0.30±0.01
Benzaldehyde	0.20±0.04
Sulcatone	0.23±0.03

### Electroantennography (EAG)

**Fig 1:**
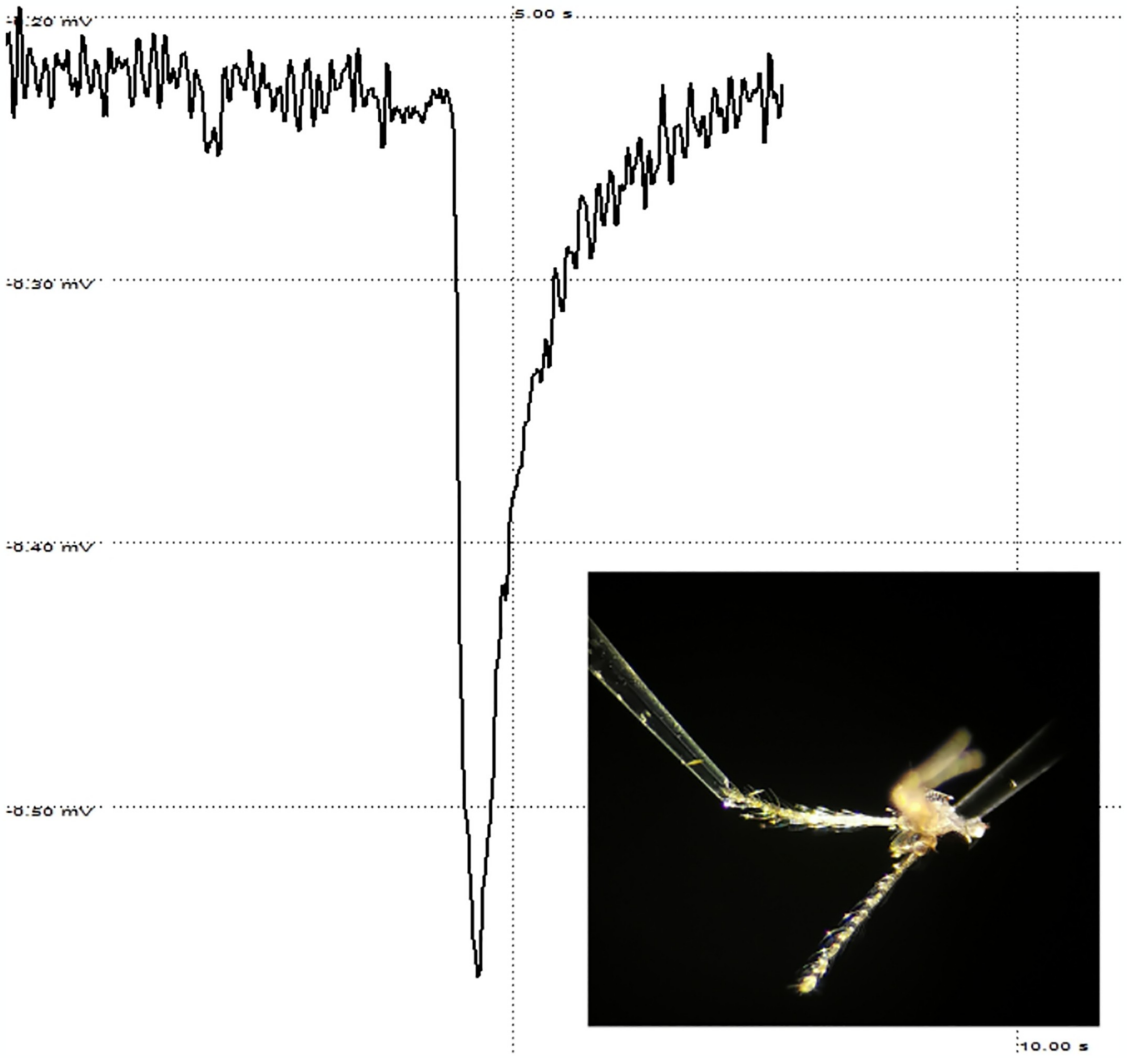
A typical electroantennogram (EAG) recording from *Phlebotomus perniciosus*; on the right a detail of the electrodes connected with insect’s antenna and head.

The chemoreceptivity of female *P*. *perniciosus* antennae to the selected VOCs was examined using the EAG technique described in previous studies [30,31]. Briefly, the head of the insect was dissected, and the distal 2–3 antennal segments were removed. A glass capillary filled with Kaissling saline [32] that served as the indifferent electrode was gently inserted into the base of the head. The tip of the amputated antenna was put in contact with the end of a similar capillary (ca. 0.1 mm diameter) which provided the recording electrode (Fig 1). Glass capillaries (Microglass, Naples, Italy) were properly pulled using a PC-10 puller (Narishige, Tokyo, Japan). AgCl-coated silver wires were used to maintain the electrical continuity between the antennal preparation and an IDAC-4 amplifier (Syntech Laboratories, Hilversum, The Netherlands) connected to a PC equipped with the EAG Pro program (Syntech Laboratories, Hilversum, The Netherlands). Before the experiment, 20 μL of each test solution was adsorbed onto a filter paper strip (1.5 cm^2^, Whatman No. 1) placed in a Pasteur pipette (15 cm long), which served as an odour cartridge. Stimuli were blown by a disposable syringe into a constant stream of charcoal-filtered humidified air (350 mL/min) flowing in a stainless-steel delivery tube (1 cm diameter) with the outlet positioned at approximately 1 cm from the antenna. Over 1 second, 2.5 cm^3^ of vapor from an odour cartridge was added. Stimuli were randomly selected whereas they were applied in ascending dose (10 μL of hexanal mineral oil solutions from 0.01 to 200 μg/μL) in dose-response experiments. Control (20 μL mineral oil) and reference (10 μL of 100 μg/μL hexanal mineral oil solution) stimuli were also applied at the beginning of the experiment and after each group of 3–4 test stimuli. Intervals between stimuli were 60 s. For each compound, EAG responses were recorded from five antennae of different females.

### Y-tube behavioural assays

The behavioural response of female *P*. *perniciosus* to VOCs selected based on their EAG activity (i.e., EAG amplitudes ≥0.30 mV) and belonging to different chemical classes, was assessed in Y-tube olfactometer bioassays. A Y-tube olfactometer consisting of a Plexiglas unit (200 × 190 × 10 mm) with a central tube (65 mm long, 15 mm large) starting from a specimen release chamber (30 mm Ø) and two lateral arms (75 mm long, 15 mm large) was used for the assays, following the protocol previously described [33,34]. A sieve inlay in the lateral arms and extending glass tube 5.25 cm away from the connection prevented the escape of insects and served as an end point of each lateral arm. The top of the unit was covered with a removable panel of glass. Humidified and medically pure air from a cylinder was passed into the extending glass tube through a Teflon connection at 0.5 mL/min. The Y-tube olfactometer was positioned horizontally, at a height of 80 cm to the ground [34]. Illumination was provided by vertically hanging red light (35 w, Trixie, Heimtierbedarf, Tarp, Germany) above (height 60 cm) the olfactometer unit. Pure compounds of the most active molecules identified by EAG were used and female sand flies were tested for each VOC, until the completion of 30 individuals making a choice (S2 Table). Each compound was delivered with a volume of 2.5 μL in a rubber device inside a Drechsel bottle connected to the Y-tube. A time of 180 s was set once the individual female was inside the olfactometer. Sand flies showing a no choice (NC) parameter (i.e., not performing a choice to one of the arms) within this period were not included within the 30 individuals tested (S2 Table). Once the sand fly entered the treatment (i.e., individual VOC) or control (i.e., ultra-pure air flow) arm of the Y-tube, a time of 20 s was set as minimum for the choice to be considered.

For each replicate a female sand fly (24 h-48 h, sugar starved, age 6–12 days) was placed inside the Y-tube trough a plastic releasing chamber. After every ten replicates, the rubber containing the substance was removed from the apparatus and replaced. To eliminate any positional bias in the room, the whole apparatus, including the Y-tube, was rotated through 180°, so that left and right sides were exchanged. To avoid any potential contamination and odour buildup, the Plexiglas unit and the glass panels of the olfactometer were wiped with hexane, washed with warm water and mild soap, rinsed with hot water for about 30 s, followed by distilled water, and, finally, dried between each rotation procedure.

### Data analysis

The EAG response was measured as the maximum amplitude of negative polarity deflection (-mV) induced by a stimulus [35]. To compensate for solvent and mechanosensory artifacts, the absolute EAG response (mV) to each test stimulus was subtracted by the mean response to the two nearest mineral oil controls [36]. To compensate for the decrease of the antennal responsiveness during the experiment, the resultant EAG value was further corrected based on the reduction of the EAG amplitude to the reference stimulus [37]. The corrected EAG responses to each compound were compared to “0” value using the Wilcoxon rank sum test to verify their measurability (*p* < 0.05). Then the Kruskal-Wallis test was used to highlight significant difference (*p* < 0.05) among the EAG responses of female sand flies to the same dose (2 mg) of the different compounds tested. In EAG dose-response experiments, the first dose at which the mean response was higher than a “0” value using the Shapiro-Wilk test for normality followed by the one-sample Student’s t-test (*p* < 0.05) was regarded as the activation threshold [38], whereas the lowest dose at which the mean response was equal to or less than the previous dose was taken as the saturation one [39].

For the behavioural tests, a likelihood χ^2^ test with Yates’ correction (*p* < 0.05) was used to compare the number of sand flies choosing the treatment arm *vs* the blank control arm of the Y-tube olfactometer. For each compound an attraction index (AI) was calculated according to Jones et al. [40].

$$\text{AI}=\frac{\text{T}-\text{C}}{\text{T}+\text{C}}$$

where T is the number of sand flies in treatment arm, C is the number of sand flies in control arm. The rate of unresponsiveness was calculated by using the proportion of sand flies that did not perform a choice in the Y-tube olfactometer by the total sand flies used for each compound. The time-in-arm was defined as the amount of time spent in a certain arm of the olfactometer during the initial selection of each sand fly. Each chemical was examined independently using a linear regression model, with the "compound" itself serving as a predictor variable. The "car" package [41] was then used to determine the significant effect of model components on the dependent variable. We then utilized the "emmeans" package [42] to do a post-hoc analysis. The analysis was done using R Statistical Software (v4.1.2; R Core Team 2021).

## Results

### Effect of dose on the EAG response

**Fig 2:**
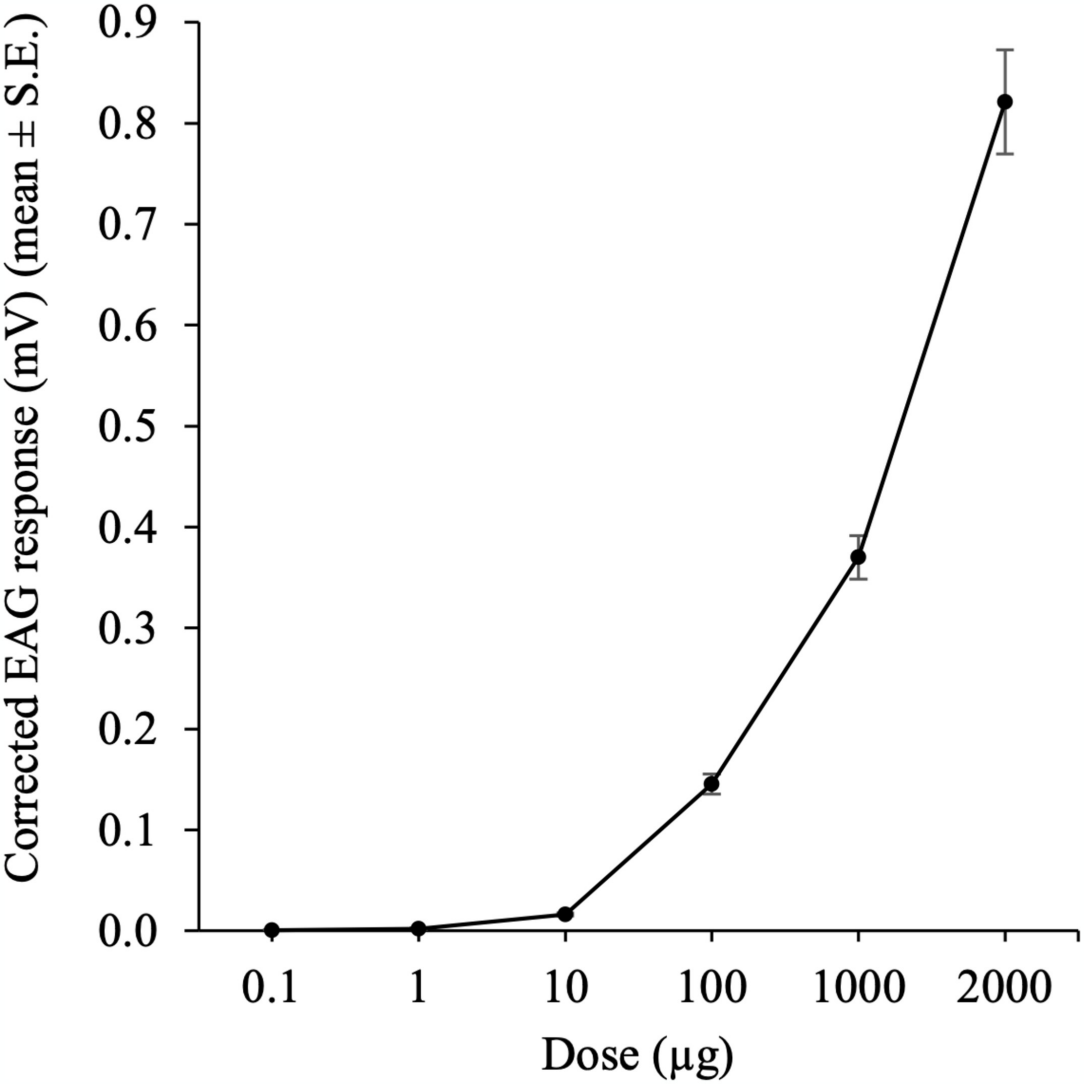
Mean (±SE) EAG dose-response curve of female *Phlebotomus perniciosus* antennae to ascending doses of hexanal.

The sensitivity of female *P*. *perniciosus* antennae to increasing doses of hexanal is shown in Fig 2. In the range of dose tested, the mean EAG response in females ranged from 0.001 ± 0.004 mV to 0.821 ± 0.051 mV. The activation threshold was 10 μg (*p* < 0.05; one-sample *t-*test). The EAG response increased from the 1000 to 2000 μg dose indicating that no saturation occurred at the 1000 μg dose.

### Antennal response pattern

The mean EAG responses of female *P*. *perniciosus* antennae to the 2000 μg dose of test VOCs are reported in Table 1. All compounds elicited measurable EAG responses (*p* < 0.05 in all Wilcoxon rank sum tests) ranging from 0.175 ± 0.019 mV (3-octanol) to 0.960 ± 0.066 mV (hexanal). Kruskal-Wallis test revealed significant differences (*H* = 113.781; *df* = 27; *p* < 0.001) among the EAG responses to different test stimuli. The largest EAG amplitudes (≥ 0.30 mV) were elicited by acetic acid, nonanoic acid, 2-propanol, 2-butanol, pentanal, hexanal, nonanal, *trans*-2-nonenal, decanal, myrcene, *p*-cymene, verbenone, 2-ethyl-1-hexanol, and acetonitrile. The weakest antennal stimulants (≤ 0.20 mV) were benzaldehyde and 3-octanol.

### Behavioural response to individual VOCs

**Fig 3:**
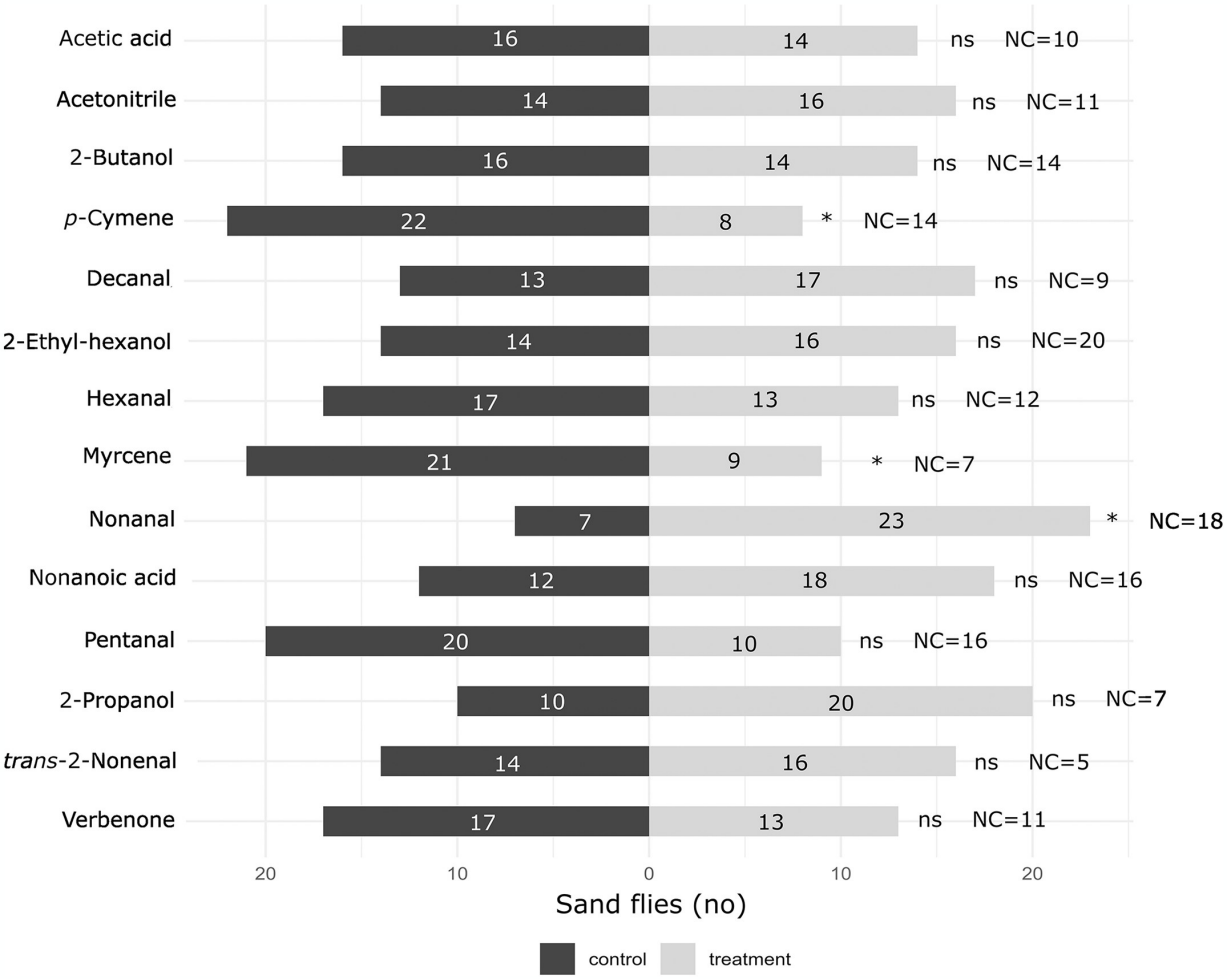
Behavioural responses of *Phlebotomus perniciosus* females to various volatile organic compounds assessed using a Y-tube olfactometer. The number of individuals who made that choice is reported within the bar. * = a significant difference was observed (*χ*^*2*^ test with Yates’ correction, *p* < 0.05); ns = not significant; NC represents the number of unresponsive females.

**Fig 4:**
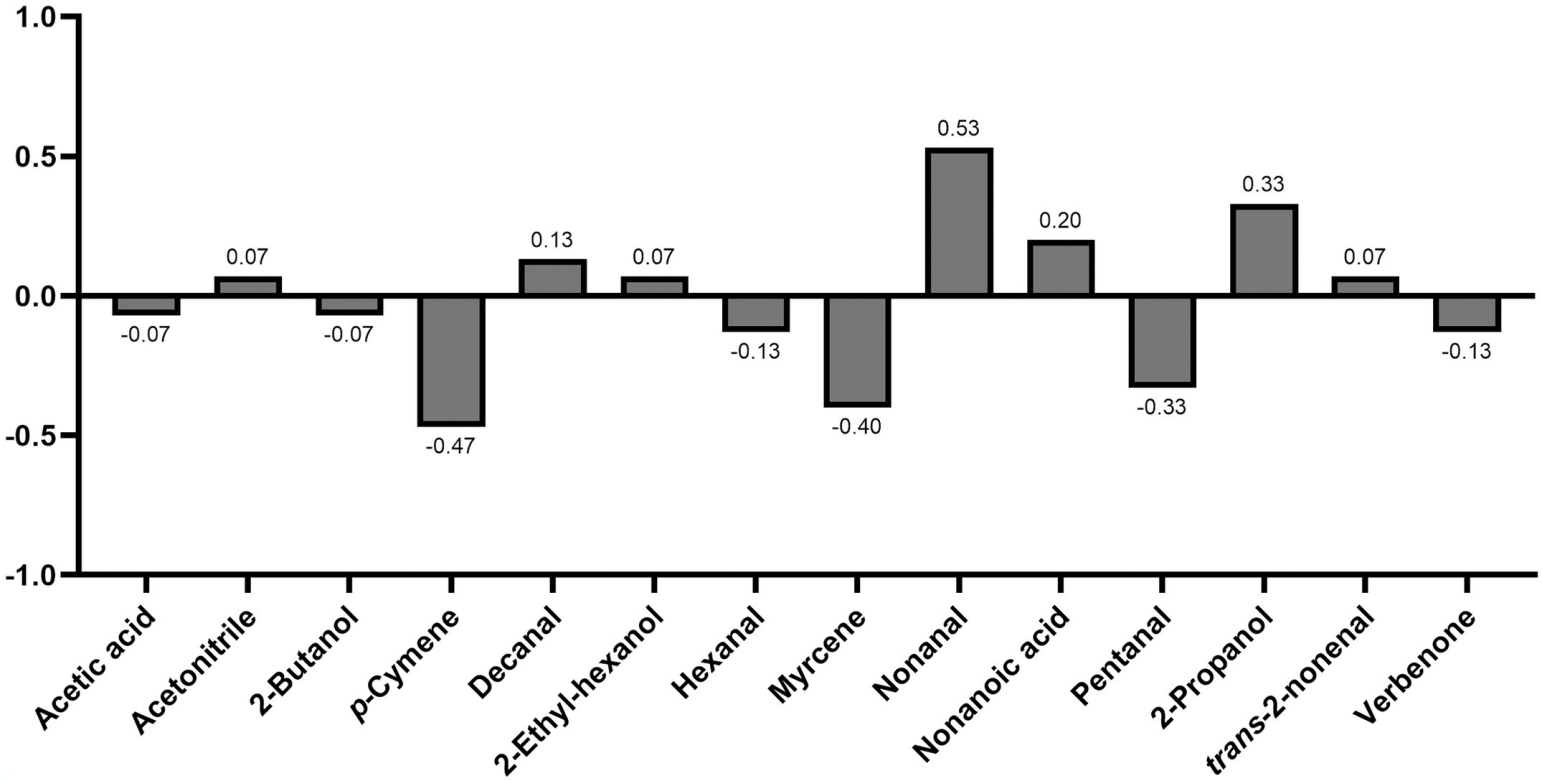
Attraction index (AI) of volatile organic compounds to *Phlebotomus perniciosus*. AI vary from -1 to 1, where -1 represents total repellence and 1 total attractiveness.

**Fig 5:**
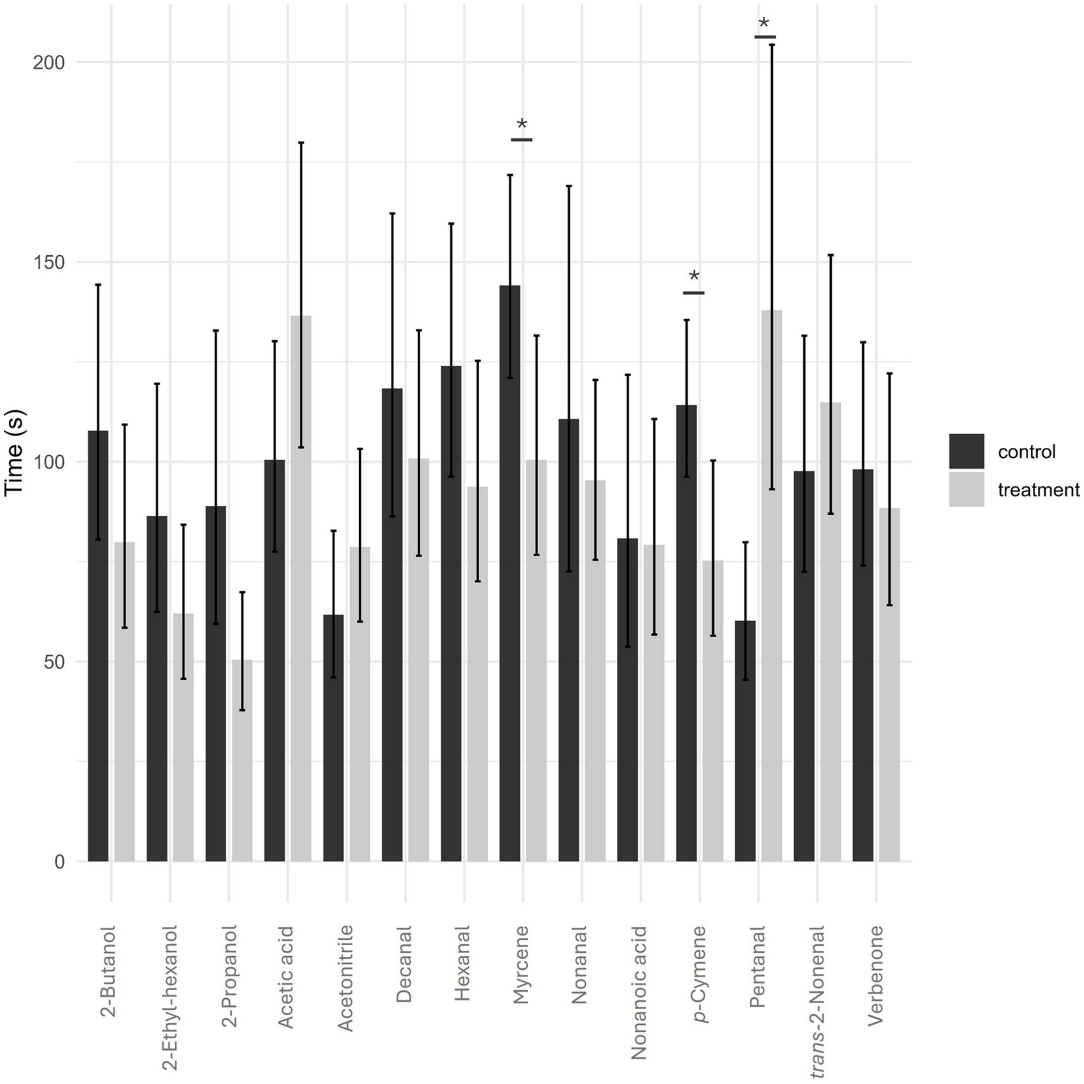
Bar plot illustrating the average (±SE) time spent by *Phlebotomus perniciosus* females in each arm of the olfactometer for the various volatile organic compounds. The minimum duration recorded was 0 s, while the maximum duration reached 180 s, marking the conclusion of the observation period. GLMM results are presented below each compound. * = a significant difference was observed (*p* < 0.05).

At the behavioural assays with Y-tube olfactometer, the compound nonanal demonstrated to be attractive for *P*. *perniciosus* females (*χ*^*2*^ = 8.53; *df* = 1; *p* < 0.05), whereas the compounds myrcene (*χ*^*2*^ = 4.80; *df* = 1; *p* < 0.05) and *p*-cymene (*χ*^*2*^ = 6.53; *df* = 1; *p* < 0.05) presented a significantly repellent activity. The other tested compounds did not elicit significant attractiveness or repellence responses in sand flies (*p* > 0.05) (Fig 3; S3 Table). The attraction indexes varied from 0.53 for nonanal (i.e., the most attractive) to -0.47 to *p*-cymene (i.e., the most repellent) VOCs (Fig 4). The rate of unresponsiveness of *P*. *perniciosus* to the VOCs tested ranged from 14.3% to 40.0% (Fig 3). The average time-in-arm varied among the several compounds tested and ranged from 74.6 to 144.1 s for the control arm, and from 64.5 to 147.0 s for the treatment arm (Fig 5). In addition, *P*. *perniciosus* spent longer time in the control arm when the compounds *p*-cymene (control vs. treated: *SE* = 16.9; *t*.*ratio* = 2.309; *p* = 0.0285) and myrcene (control vs. treated: *SE* = 16.6; *t*.*ratio* = 2.632; *p* = 0.0139) were tested. Sand flies spent longer time in the pentanal-treated arm (control vs. treated: *SE* = 21.2; *t*.*ratio* = -3.254; *p* = 0.0030) (Fig 5).

## Discussion

Our study identified nonanal as an attractive VOC for *P*. *perniciosus* females, while myrcene and *p*-cymene elicited a repellent response, as outlined by first choice and time-in-arm data analysis. These data are unprecedented for sand fly species of the genus *Phlebotomus*, as previous studies on VOCs causing attractive response were only assessed for species within the subgenera *Lutzomyia* and *Nyssomyia*, either by testing compounds individually or as blends [14–21]. While the results clearly demonstrate the attractiveness of nonanal and the repellence of myrcene and *p*-cymene to *P*. *perniciosus*, further investigations are warranted to explore the potential synergy of these VOCs with other compounds and their effects on other sand fly species.

Nonanal has been found attractive for *Culex quinquefasciatus* Say, 1823 in field experiments [43], and has also been demonstrated to be one of the most potent antennal stimuli for *Anopheles gambiae* Giles, 1902 mosquitoes [44]. In addition, it was detected in high levels in humans infected with *Plasmodium falciparum* Welch, 1897, the causative agent of malaria, being consequently regarded as a marker of infection by this protozoan [45]. Similarly, nonanal was selectively expressed in *Leishmania*-infected vs non-infected dogs, with higher abundance in infected individuals, suggesting its potential as a biomarker of *L*. *infantum* infection in dogs [28]. This could explain the observed higher attraction of sand fly vectors towards dogs infected with *L*. *infantum* [22], potentially facilitating parasite transmission and perpetuation in vertebrate hosts.

The attractiveness of *P*. *perniciosus* to nonanal under laboratory conditions also suggests that this compound is a promising option for use as an olfactory cue to enhance the effectiveness of sand fly traps. However, field studies are needed to assess its potential as an attractant for this sand fly species. Indeed, nonanal significantly increased the number of *Cx*. *quinquefasciatus* mosquitoes captured using EVS traps (Bioquip) in field trials [43]. In addition, in the same study nonanal synergized with CO_2_, leading to a 50% increase in mosquitoes capture, compared to traps using CO_2_ alone [43].

A repellent response was observed in *P*. *perniciosus* females exposed to the terpenes myrcene and *p*-cymene, both of which are present in plants and essential oils [46], as well as on the skin of animals, such as dogs [29]. Interestingly, both myrcene and *p*-cymene were detected through GC-MS in dogs negative for *Leishmania* spp. [29], but not in infected ones [28]. This suggests that these naturally produced compounds may reduce the number of sand fly bites, yet the infection with *Leishmania* spp. Additionally, both terpenes have been shown to repel *Aedes aegypti* Linnaeus, 1762 by inhibiting the blood feeding in 47% and 57% of the mosquitoes exposed to *p*-cymene and to myrcene, respectively [47]. Moreover, evaluating the production of these terpenes in different dog breeds is advocated to understand whether there is an effect on the attractivity for sand flies, as it has previously demonstrated for *Rhipicephalus sanguineus* sensu lato Latreille, 1806 ticks [48,49].

Apart from nonanal, myrcene and *p*-cymene, other VOCs, particularly pentanal, hexanal, *trans*-2-nonenal, decanal, 2-propanol, 2-butanol, 2-ethyl-1-hexanol, acetic acid, nonanoic acid, verbenone, and acetonitrile triggered strong antennal response in sand flies, indicating that these compounds may affect *P*. *perniciosus* behaviour; however, they showed no significant effects in olfactometer assays. The lack of a significant olfactory response of *P*. *perniciosus* towards the above compounds could be associated with factors such as the concentration of the compounds (i.e., in this study only pure compounds were used), and/or time needed for sand fly activation when exposed to these molecules. Therefore, further studies exploring different concentrations and exposure conditions of sand flies to the above VOCs are advocated. This data also indicates that while the EAG is a useful tool for measuring the physiological response of insects to VOCs, it does not reveal whether these compounds are attractive or repellent. For example, in a study performed with the spotted asparagus beetle, *Crioceris duodecimpunctata* (L.), the compound (*Z*)-3-hexen-1-ol was the most EAG-active compound; however, at Y-tube behavioural assays the same compound did not elicit any response to females, but for males it was attractive at a dose of 10 μg and repellent at a dose of 50 μg [31]. The above suggests that combining electrophysiology with behavioural assays (i.e., olfactometer) is essential for a comprehensive understanding of both detection and behavioral responses to VOCs in host-seeking behavior.

## Conclusions

Results herein reported demonstrated that laboratory-reared *P*. *perniciosus* sand flies are attracted to nonanal, an important compound present in *L*. *infantum-*infected dogs. These finding shed light on the development of sustainable sand fly monitoring and control in leishmaniasis endemic areas. Moreover, myrcene and *p*-cymene were found to repel this sand fly species, making them potential candidates for use as repellents. Data provided in this study are also unprecedented for sand flies of the genus *Phlebotomus*, and the effect of VOCs on the olfactory response of other species of Old World sand flies deserves to be investigated. Furthermore, field trials are recommended to assess whether nonanal can enhance the efficiency of sand fly traps, providing further understanding of its effect on sand fly behaviour.

## Supporting information

S1 Table List of Volatile Organic Compounds identified in humans, *Leishmania* infected, and non-infected dogs in previous studies [18,27–29]. (DOCX)

S2 Table Number of *Phlebotomus perniciosus* used at the behavioural assays with the Y-tube olfactometer. (DOCX)

S3 Table χ^2^ test with Yates’ correction (*p* = 0.05) comparing the number of *Phlebotomus perniciosus* choosing the treatment over the blank control arm of the Y tube olfactometer. (DOCX)
