# Supplementary material for: Phlebotomus perniciosus response to volatile organic compounds of dogs and humans
Source: PLoS Negl Trop Dis. 2024 Dec 30;18(12):e0012787. doi: 10.1371/journal.pntd.0012787 (PMC11723633; doi:10.1371/journal.pntd.0012787)
Supplement: S1 Table — (DOCX) [file pntd.0012787.s001.docx]

**S1 Table.** List of Volatile Organic Compounds identified in humans, *Leishmania* infected, and non-infected dogs in previous studies [18, 27-29]

|  | **Humans** | **Infected dogs** | **Non-infected dogs** |
| --- | --- | --- | --- |
|  | 6-methylhept-5-en-2-one |  |  |
|  | Octanal | Octanal | Octanal |
|  | 2-phenylacetaldehyde |  |  |
|  | 1-octanol |  |  |
|  | 6-methyl-3,5-heptadien-2-one |  |  |
|  | Nonanal | Nonanal |  |
|  | Cis-verbenol |  |  |
|  | 2-nonenal | 2-nonenal |  |
|  | 1-nonanol |  |  |
|  | Verbenone |  |  |
|  | Decanal | Decanal |  |
|  | 2-decenal | 2-decenal |  |
|  | Nonanoic acid |  |  |
|  | Undecanal | Undecanal |  |
|  | 2-undecenal |  |  |
|  | Dodecanal | Dodecanal |  |
|  | Tetradecane | Tetradecane | Tetradecane |
|  | Gamma decalactone |  |  |
|  | Methylparaben |  |  |
|  | Geranylacetone |  | Geranylacetone |
|  | Dodecen-1-al |  |  |
|  | 1-dodecanol | 1-dodecanol |  |
|  | Tridecanal |  |  |
|  | Pentadecane | Pentadecane |  |
|  | (E)-2-tridecenal |  |  |
|  | Dodecanoic acid |  |  |
|  | Tetradecanal |  |  |
|  | Hexadecane | Hexadecane |  |
|  | 1-tetradecanol |  |  |
|  | Pentadecanal |  |  |
|  | Heptadecane | Heptadecane |  |
|  | Tetradecanoic acid | Tetradecanoic acid |  |
|  | 2-ethylhexyl salicylate | 2-ethylhexyl salicylate |  |
|  | 2-phenyl dodecane |  |  |
|  | Octadecane | Octadecane |  |
|  | Pentadecanoic acid |  |  |
|  | 1-hexadecanol |  |  |
|  | Nonadecane | Nonadecane |  |
|  | Hexadecanoic acid |  |  |
|  | Icosane |  |  |
|  | Isopropil palmitate |  |  |
|  | 1-octadecanol |  |  |
|  |  | Heptanal | Heptanal |
|  |  | Benzaldehyde | Benzaldehyde |
|  |  | Decane | Decane |
|  |  | Acetophenone |  |
|  |  | Octanol |  |
|  |  | Heptanoic acid |  |
|  |  | Undecane |  |
|  |  | Naphtalene |  |
|  |  | 2-Decanone |  |
|  |  | β-hydroxyethyl phenyl ether |  |
|  |  | Dodecano |  |
|  |  | 2-Undecanone |  |
|  |  | Tridecane |  |
|  |  | Nonyl-cyclopentane |  |
|  |  | 2,6-Bis (1,1-dimethylethyl-4-methyl)-phenol |  |
|  |  | Benzophenone |  |
|  |  | 3-Methyl-pentadecane |  |
|  |  | 8-Pentadecanone |  |
|  |  | Octyl ether |  |
|  |  |  | Acetonitrile |
|  |  |  | Propanone |
|  |  |  | Isopropyl alcohol |
|  |  |  | Acetic acid |
|  |  |  | Ethyl ester |
|  |  |  | Butanal, 3methyl |
|  |  |  | 1-Butanol |
|  |  |  | Pentanal |
|  |  |  | Propane, 1,2-dichloro |
|  |  |  | Heptane |
|  |  |  | Hexanal |
|  |  |  | 1-Pentanol |
|  |  |  | Toluene |
|  |  |  | Hexanal |
|  |  |  | Furfural |
|  |  |  | 4-Hydroxy-4-methyl-2-pentanone |
|  |  |  | Isovaleric Acid |
|  |  |  | Xylene |
|  |  |  | Valeric Acid |
|  |  |  | 2-Butoxyethanol |
|  |  |  | α-Pinene |
|  |  |  | 6-Methyl-5-heptene-2-one |
|  |  |  | a-Myrcene |
|  |  |  | 4-Cyanocyclohexene |
|  |  |  | Benzyl Alcohol |
|  |  |  | 2-Ethyl-1-hexanol |
|  |  |  | D-Limonene |
|  |  |  | p-Cymene |
|  |  |  | Menthol |
