# Supplementary material for: Phlebotomus perniciosus response to volatile organic compounds of dogs and humans
Source: PLoS Negl Trop Dis. 2024 Dec 30;18(12):e0012787. doi: 10.1371/journal.pntd.0012787 (PMC11723633; doi:10.1371/journal.pntd.0012787)
Supplement: S2 Table — (DOCX) [file pntd.0012787.s002.docx]

| **Choice** | **Pentanal** | **Hexanal** | **Nonanal** | ***trans*-2-nonenal** | **Decanal** | **2-Propanol** | **2-Butanol** | **2-Ethyl-1-hexanol** | **Acetic acid** | **Nonanoic acid** | **Myrcene** | ***p*-Cymene** | **Verbenone** | **Acetonitrile** |
| --- | --- | --- | --- | --- | --- | --- | --- | --- | --- | --- | --- | --- | --- | --- |
| **T** | 10 | 13 | 23 | 16 | 17 | 20 | 14 | 16 | 14 | 18 | 9 | 8 | 13 | 16 |
| **C** | 20 | 17 | 7 | 14 | 13 | 10 | 16 | 14 | 16 | 12 | 21 | 22 | 17 | 14 |
| **NC** | 16 | 11 | 18 | 5 | 9 | 7 | 14 | 20 | 10 | 16 | 7 | 14 | 11 | 11 |

**S2 Table.** Number of *Phlebotomus perniciosus* used at the behavioural assays with the Y-tube olfactometer.

T: treatment; C: control; NC: No choice
