# Supplementary material for: Phlebotomus perniciosus response to volatile organic compounds of dogs and humans
Source: PLoS Negl Trop Dis. 2024 Dec 30;18(12):e0012787. doi: 10.1371/journal.pntd.0012787 (PMC11723633; doi:10.1371/journal.pntd.0012787)
Supplement: S3 Table — (DOCX) [file pntd.0012787.s003.docx]

| **Choice** | **Pentanal** | **Hexanal** | ***Nonanal** | ***trans*-2-nonenal** | **Decanal** | **2-Propanol** | **2-Butanol** | **2-Ethyl-1-hexanol** | **Acetic acid** | **Nonanoic acid** | ***Myrcene** | ***p-Cymene** | **Verbenone** | **Acetonitrile** |
| --- | --- | --- | --- | --- | --- | --- | --- | --- | --- | --- | --- | --- | --- | --- |
| ***χ^2^*** | 3.333^a^ | 0.533^a^ | 8.533^a^ | 0.133^a^ | 0.533^a^ | 3.333^a^ | 0.133^a^ | 0.133^a^ | 0.133^a^ | 1.200^a^ | 4.800^a^ | 6.533^a^ | 0.533^a^ | 0.133^a^ |
| **df** | 1 | 1 | 1 | 1 | 1 | 1 | 1 | 1 | 1 | 1 | 1 | 1 | 1 | 1 |
| ***p*-value** | 0.068 | 0.465 | 0.003* | 0.715 | 0.465 | 0.068 | 0.715 | 0.715 | 0.715 | 0.273 | 0.028 | 0.011 | 0.465 | 0.715 |

**S3 Table.** χ^2^ test with Yates’ correction (*p* = 0.05) comparing the number of *Phlebotomus perniciosus* choosing the treatment over the blank control arm of the Y tube olfactometer.

1. 0 cells (0.0%) have expected frequencies less than 5. The minimum expected cell frequency is 15.0. *Statistically significant
